# Secretion of Protective Antigens by Tissue-Stage Nematode Larvae Revealed by Proteomic Analysis and Vaccination-Induced Sterile Immunity

**DOI:** 10.1371/journal.ppat.1003492

**Published:** 2013-08-15

**Authors:** James P. Hewitson, Al C. Ivens, Yvonne Harcus, Kara J. Filbey, Henry J. McSorley, Janice Murray, Stephen Bridgett, David Ashford, Adam A. Dowle, Rick M. Maizels

**Affiliations:** 1 Institute of Immunology and Infection Research, Ashworth Laboratories, School of Biological Sciences, University of Edinburgh, Edinburgh, United Kingdom; 2 Centre for Immunity, Infection and Evolution, Ashworth Laboratories, School of Biological Sciences, University of Edinburgh, Edinburgh, United Kingdom; 3 Gene Pool, Ashworth Laboratories, School of Biological Sciences, University of Edinburgh, Edinburgh, United Kingdom; 4 Technology Facility, University of York, York, United Kingdom; National Institutes of Health, United States of America

## Abstract

Gastrointestinal nematode parasites infect over 1 billion humans, with little evidence for generation of sterilising immunity. These helminths are highly adapted to their mammalian host, following a developmental program through successive niches, while effectively down-modulating host immune responsiveness. Larvae of *Heligmosomoides polygyrus*, for example, encyst in the intestinal submucosa, before emerging as adult worms into the duodenal lumen. Adults release immunomodulatory excretory-secretory (ES) products, but mice immunised with adult *H. polygyrus* ES become fully immune to challenge infection. ES products of the intestinal wall 4th stage (L4) larvae are similarly important in host-parasite interactions, as they readily generate sterile immunity against infection, while released material from the egg stage is ineffective. Proteomic analyses of L4 ES identifies protective antigen targets as well as potential tissue-phase immunomodulatory molecules, using as comparators the adult ES proteome and a profile of *H. polygyrus* egg-released material. While 135 proteins are shared between L4 and adult ES, 72 are L4 ES-specific; L4-specific proteins correspond to those whose transcription is restricted to larval stages, while shared proteins are generally transcribed by all life cycle forms. Two protein families are more heavily represented in the L4 secretome, the Sushi domain, associated with complement regulation, and the ShK/SXC domain related to a toxin interfering with T cell signalling. Both adult and L4 ES contain extensive but distinct arrays of Venom allergen/*Ancylostoma* secreted protein-Like (VAL) members, with acetylcholinesterases (ACEs) and apyrase APY-3 particularly abundant in L4 ES. Serum antibodies from mice vaccinated with L4 and adult ES react strongly to the VAL-1 protein and to ACE-1, indicating that these two antigens represent major vaccine targets for this intestinal nematode. We have thus defined an extensive and novel repertoire of *H. polygyrus* proteins closely implicated in immune modulation and protective immunity.

## Author Summary

Intestinal helminth parasites are highly prevalent in humans and animals, and survive for long periods by deviating the host immune system. No vaccines are currently available to control these infections. Many helminths invade through barrier surfaces (such as the skin or the digestive tract) and develop through tissue larval stages before reaching their final niche such as the intestinal lumen. We studied the tissue larval stage of a mouse parasite, *Heligmosomoides polygyrus*, to test whether proteins released by this stage could elicit protective immunity, and found that they indeed constitute very effective vaccine antigens. Proteomic analysis to identify the individual proteins released by the larvae demonstrated that while many products are shared between tissue-dwelling larvae and adults occupying the intestinal lumen, larvae express higher levels of two gene families linked to immunomodulation, namely the Sushi protein family and the ShK toxin family. Antibody analysis of serum from vaccinated mice identified two major antigens recognised by the protective immune response as VAL-1 and ACE-1, which are respectively members of the venom allergen and acetylcholinesterase families. This work establishes that tissue larvae are the source of protective antigens for future vaccines, and highlights their production of two potentially immunomodulatory gene families.

## Introduction

Gastro-intestinal nematode parasites are among the most prevalent pathogens in the world, afflicting over 1 billion people [1] and causing widespread disease in livestock [2]. Control through drug therapy is compromised by rapid reinfection [3], reflecting the lack of protective immunity generated during natural exposure. Helminth infection is also associated with a wide suite of immunological down-modulatory effects [4], which have evolved to promote parasite survival. Although it is possible to vaccinate animals against helminthiases, few protective antigens have been defined and immunity reduces rather than eliminates worm loads [1], [5].

*Heligmosomoides polygyrus* is an ideal model species to study host-parasite interactions in gastro-intestinal helminth infection [6]–[8]. The parasite follows an entirely intestinal course of infection, entering orally, developing through larval stages before establishing as long-lived adults in the lumen of the small intestine, releasing eggs that are transmitted through feces for onward transmission. Infected mice show multiple immuno-modulatory changes including expansion of regulatory T cells [9]–[12], B cells [13] and dendritic cells [14]–[16]. The immunoregulatory environment engendered by *H. polygyrus* extends to dampening bystander immune responses to allergens, autoantigens and intestinal antigens [4], [17], [18].

A critical point in infection is during the first 8–10 days, when incoming larvae invade the intestinal tissue and become encysted in the submucosa adjacent to the serosal membrane. Depending on the genetic background of the murine host, a localised immune reaction can envelop the larva, forming a macrophage-rich granuloma. In primary infection however, parasites can escape these inflammatory foci and successfully migrate to the lumen to continue their life cycle [19].

How parasites evade immune attack in the tissues has yet to be determined, but it is known that systemic immune suppression is associated not only with the long-lived adult stage but also the larval stage in the intestinal wall [20]. Moreover, mice given curative anthelminthic treatment prior to adult maturation [21], or heavily irradiated infective larvae [22] develop protective immunity to challenge infection. Hence, as well as being a source of protective antigens, immature tissue-dwelling parasites actively contribute to immunological down-regulation, and we hypothesise that in the non-immune setting they are able to deflect or disable immune mechanisms within the gut tissue.

The ability of parasites to modulate immunity is likely to be dependent on the molecular components secreted into their mammalian host, reproduced by the “excretory-secretory” (ES) products collected from parasites maintained *in vitro* [23]. Global proteomic analyses of the parasite “secretome” are proving to be important and illuminating steps towards defining the host-parasite interaction at the molecular level [24]. Thus, the secretome of adult *H. polygyrus* has been found to contain more than 300 proteins, including many enzymes and homologues of host immune system genes, as well as novel proteins whose precise function has yet to be defined [25], [26].

Parasite ES products have also proven to be effective vaccines in many settings [1], [5], not least in the case of *H. polygyrus* in which adult HES immunization elicits sterilizing immunity in mice [27]. An attractive hypothesis for this protective effect is that vaccination generates neutralising antibodies that counter the immunomodulatory molecules secreted from the host. If such vaccines can also be directed against the immature stage of helminths, such as the tissue-phase larvae of *H. polygyrus*, then parasites may be eliminated before egg production and transmission can be attained.

We have therefore studied the released products of *H. polygyrus* tissue phase larvae, both to identify individual protein components, and to test their ability to induce immunity through vaccination. As detailed below, this investigation identifies a shift in secreted protein composition between the tissue and luminal phases, with expansion and contraction of different gene sets, in particular in the representation of the major multi-gene protein families associated with nematode infection. Most importantly, the larval secretions are fully immunogenic, induce complete sterilising immunity, and have allowed us to identify key candidate protein antigens that may form the basis of a subunit vaccine for protective immunity.

## Results

### Secretome of 4th-stage *H. polygyrus* larvae

To identify the secretory products of the tissue-dwelling phase of *H. polygyrus*, individual L4 larvae were isolated from the submucosa of mice 5 days (120 hours) following oral infection with third-stage (L3) infective larvae, at a point soon after the third molt (90–96 hours) but prior to the fourth and final molt to the adult stage (144–166 hours) [28]. Using serum-free media for cultivation, in conditions previously reported for adult *H. polygyrus* [25], larvae were cultured for 3 days and supernatants collected, concentrated using centrifugal filters and analysed for recovered proteins. We also collected material released by *H. polygyrus* eggs (ERM; egg released material) harvested *in vitro* from adult worms collected from the lumen of infected mice to act as an outgroup as the egg stage has not previously been associated with immunity or immunomodulation.

**Figure 1:**
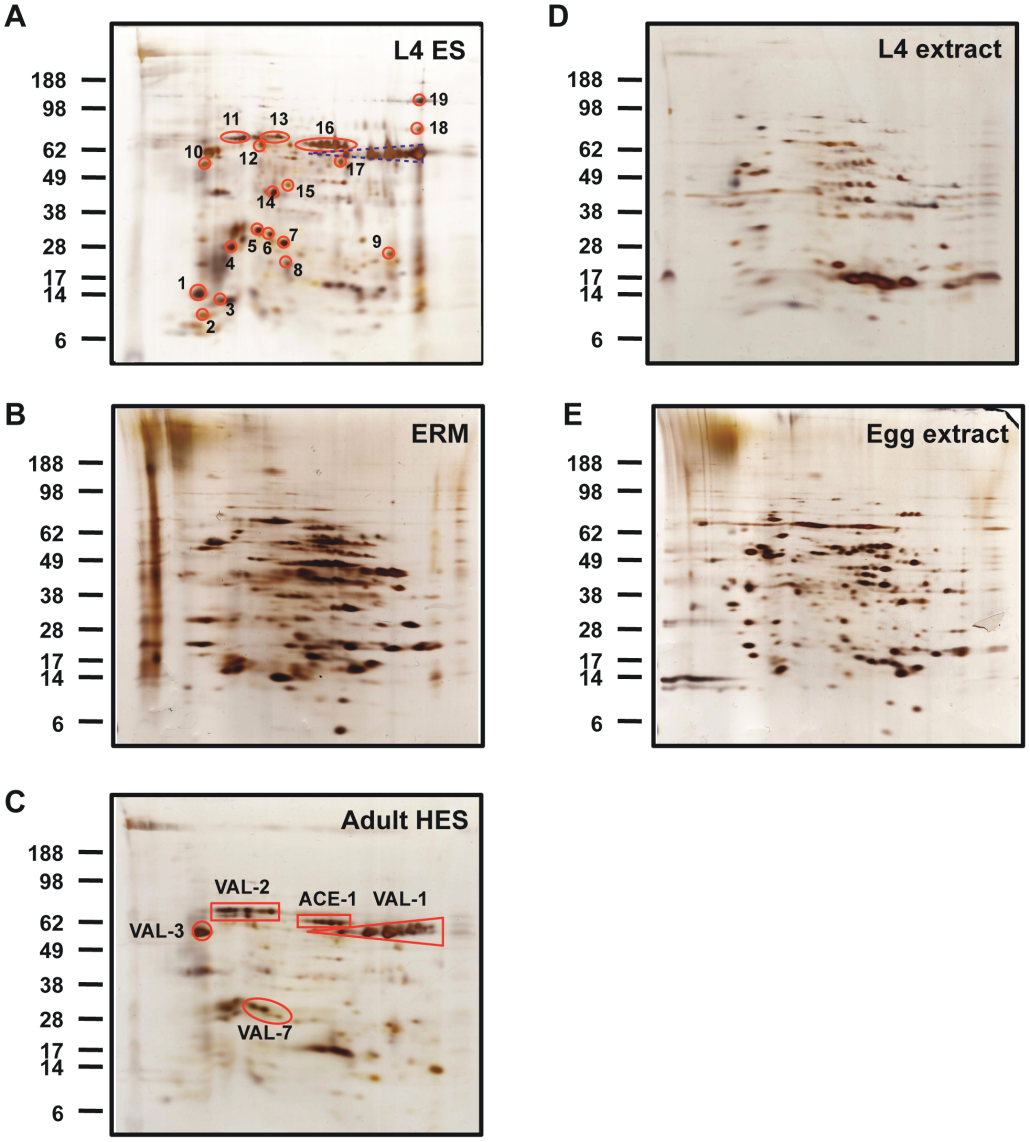
2-D gel electrophoresis of ES released material and somatic extracts from different stages of *H.polygyrus*, visualised by silver staining. **A.** L4 ES. Identities of numbered spots are given in Table 1. **B.** Egg released material (ERM). **C.** Adult HES. Solid red boxes correspond to the indicated protein products. **D.** L4 somatic extract. **E.** Egg somatic extract. Positions of molecular weight markers (kDa) are indicated.

Proteins in ES or released material from the three life cycle stages were first compared by 2-dimensional SDS-PAGE and silver staining. As shown in **Fig. 1**, L4 ES (**Fig. 1 A**) was clearly very distinct from egg-released material (ERM) (**Fig. 1 B**), but bore a number of similarities with adult HES (**Fig. 1 C**). When L4 ES was compared to somatic extract from the same stage (**Fig. 1 D**), a number of proteins enriched or only visible in the secreted fraction were apparent. In contrast, most spots appeared to be shared between ERM (**Fig. 1 B**) and egg somatic extract (**Fig. 1 E**), suggesting in this case passive release rather than selective secretion. In the comparison between L4 and adult ES, a number of co-localising spots have previously been identified in HES including members of the Venom allergen/*Ancylostoma* secreted protein-Like (VAL) family and acetylcholinesterases (ACE) [25]).

MS analysis of individual L4 ES spots, detailed in **Table 1**, confirmed the presence of two forms of ACE (ACE-1 and -2), as well as several VAL proteins, particularly variants of VAL-7, the lysozymes LYS-1 and LYS-2, apyrase APY-3, and the previously described Th2-skewing protein calreticulin [29]. In addition, proteins with no known homologs in other species were present, including variants of a Novel Secreted Protein-3 (NSP-3) found in adult HES [25] and a newly described Larval Secreted Protein (LSP) not detected in HES.

**Table 1:** L4 ES protein spot identities.

Spot	Identity	Isotig and Isogroup	Peptides	Score
1	**NSP-3.3**	Hp_I01045_IG00068 Hp_I01047_IG00068	6	464
2	**MPP-like**	Hp_I10549_IG03359 Hp_I10550_IG03359	1	98
3	**NSP-3.2**	Hp_I17967_IG09911	3	241
	**NSP-42**	Hp_I02051_IG00167	1	69
4	**Lysozyme-1**	Hp_I08665_IG02417	5	522
5	**VAL-7.4**	Hp_I01449_IG00104	6	662
	**VAL-7.3**	Hp_I01450_IG00104	6	592
6	**VAL-7.5**	Hp_I01454_IG00104	5	394
	**VAL-7.3**	Hp_I01450_IG00104	4	293
	**VAL-7.2**	Hp_I01453_IG00104	4	265
7	**VAL-7.1**	Hp_I01451_IG00104 Hp_I01452_IG00104	7	551
8	**Sushi-like**	Hp_I08843_IG02506	2	246
	**NSP-62**	Hp_I30075_IG22019	1	134
9	**Lysozyme-2**	Hp_I05758_IG01053	3	242
	**Deoxyribonuclease II**	Hp_I15874_IG07818	2	181
	**VAL-25**	Hp_I10899_IG03534 Hp_I19276_IG11220	1	78
10	**Calreticulin**	Hp_C00231_IG00001	3	213
11	**ACE-2**	Hp_I04629_IG00719	7	470
	**VAL-2.3**	Hp_I07952_IG02060	1	77
12	**VAL-9**	Hp_I14221_IG06165	8	604
13	**VAL-2.1/2.2/2.3**	Hp_I07952_IG02060 Hp_I07953_IG02060 Hp_I15068_IG07012	3	214
14	**APY-3**	Hp_I06737_IG01430	5	622
15	**Astacin protease**	Hp_I13832_IG05776	2	144
16	**ACE-1**	Hp_I12803_IG04747	6	618
17	**VAL-13**	Hp_I13967_IG05911	4	329
18	**LSP-1**	Hp_I03144_IG00345	8	664
19	**Kunitz inhibitor**	Hp_I12299_IG04234	1	165

Spot numbers refer to those in **Fig. 1 A**. One identity is shown where the same peptides match multiple isotigs (i.e. variants). Additional identities are shown where distinct peptides match either variants of the same protein or different proteins that co-localise within the same gel area. Number of unique peptides identifying each protein is shown, as is the mascot score.

### Excretory-secretory products from 4th-stage larvae, but not eggs, induce sterilising immunity to challenge infection with *H. polygyrus*

Our first objective was to test whether L4 ES antigens were able and sufficient to generate protective immunity in mice that are normally susceptible to primary infection with *H. polygyrus*. We have previously shown that vaccination with adult HES in alum adjuvant generates sterile immunity against challenge [27]. We therefore compared immunization with L4 ES, HES and egg-derived ERM.

**Figure 2:**
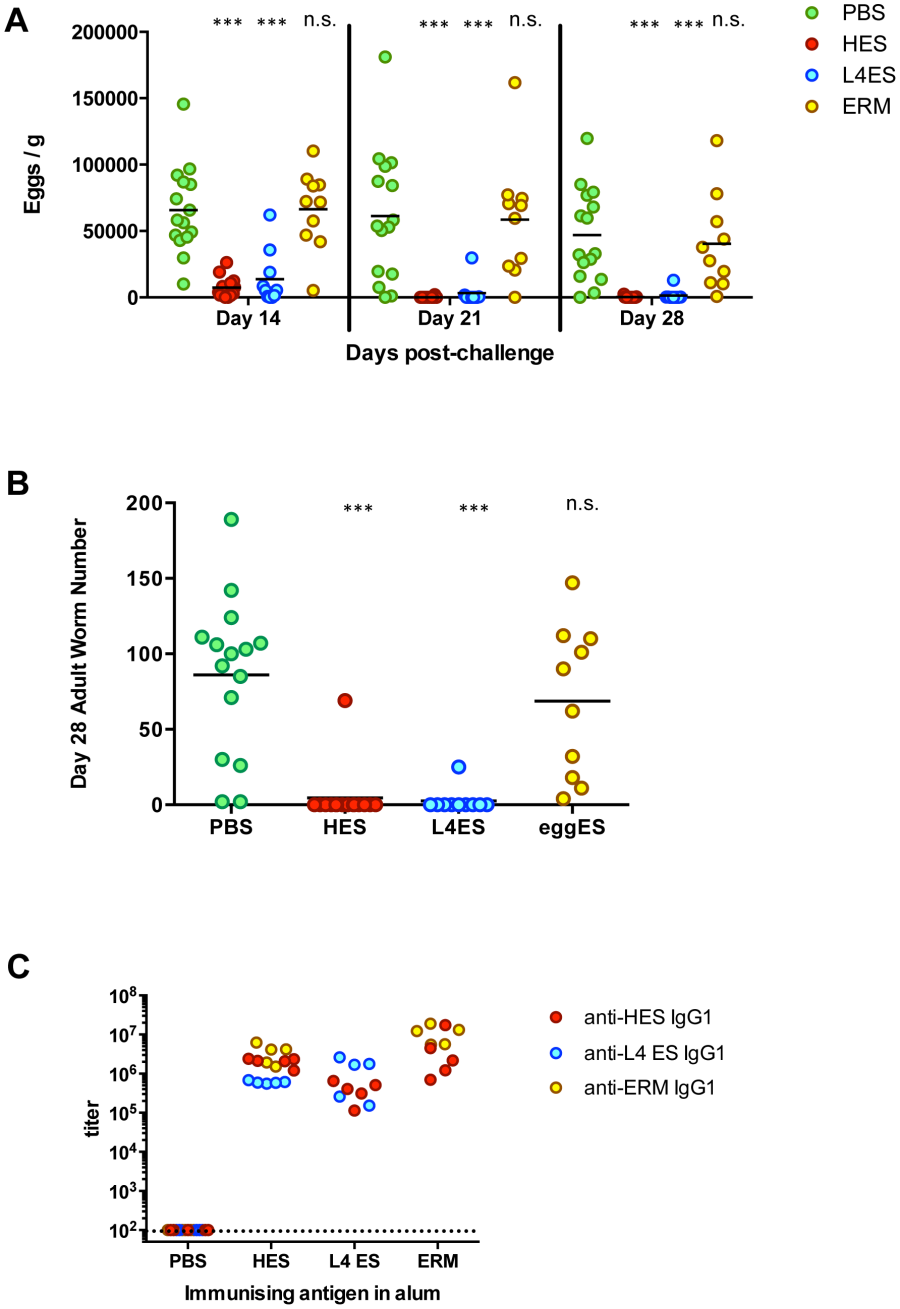
Protective immunity following L4 ES or HES, but not ERM, immunization of C57BL/6 mice. **A.** Fecal egg burdens in mice at days 14, 21 and 28 post-challenge. **B.** Adult worm recoveries at day 28 post-challenge. **C.** Anti-ES IgG1 titers in immunized mice prior to challenge. Anti-HES (red), anti-L4 ES (blue) and anti-ERM (yellow) titers are shown following HES, L4 ES or ERM immunisation. Data in (A–B) are pooled from two individual experiments with 5–10 mice per group, and data in (C) are representative of two individual experiments. Statistical significance refers to ANOVA comparisons with PBS/alum group (*** p<0.001; n.s. = non-significant).

Immunity in vaccinated mice was measured both by fecal egg output and worm burden at autopsy. Egg burdens measured between 14 and 28 days of infection rapidly approached zero in mice vaccinated with either L4 ES or HES (**Fig. 2 A**), while adult worm burdens at day 28 showed that both conferred sterile immunity on nearly all mice (**Fig. 2 B**). In contrast, ERM immunization did not affect adult worm burdens or parasite fecundity (**Fig. 2 A, B**). HES-immunized mice produced high levels of anti-HES IgG1 antibodies (**Fig. 2 C**), the isotype most important in antibody-mediated immunity following secondary infection [30]. Notably, anti-HES IgG1 titers were similar in ERM- and HES-immunized mice, showing that the failure of ERM immunization is not due to any intrinsic lack of immunogenicity (**Fig. 2 C**). Instead, this emphasised the importance of identifying specific molecular targets following protective immunisation, a process that required we first gain a deeper understanding of the individual protein components in each ES preparation.

### LC-MS identification and quantification of L4 and egg secreted proteins

**Figure 3:**
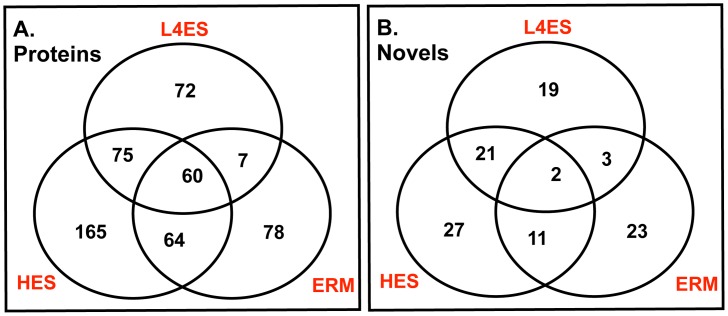
Shared and stage-specific ES proteins. **A.** Venn diagram of total secretome proteins from three stages, enumerating specific and shared identities. **B.** Venn diagram of subset of total proteins showing no database similarity to known proteins in other species. Such proteins in Adult HES (irrespective of presence in other stages) are designated as previously described [25] as Novel Secreted Proteins (NSPs), while those found in L4 ES but not the adult are Larval Secreted Proteins (LSPs).

Taking advantage of an expanded *H. polygyrus* nucleotide database based on >1 million cDNA sequences from 5 different life-cycle stages of *H. polygyrus* (L3, day 3, day 5, adult and eggs) including each of those from which the secreted proteins were collected, we subjected L4 ES and ERM to LC-MS/MS analysis and compared them to adult HES [25]. This analysis identified 214 proteins present in L4 ES, and 209 in ERM, in comparison to the secretion of 364 in adult HES. The number of proteins shared between adult and L4 (63.1% = 135/214 L4 ES proteins), and adult and ERM (59.3% = 124/209 ERM proteins) were broadly similar (**Fig. 3 A**). Both L4 (33.6% = 72/214) and ERM (37.3% = 78/209) contained a similar proportion of proteins detected only in that stage. Very few proteins (7) were shared between L4 ES and ERM but were not detected in HES, while a core of 60 ES proteins was shared between the three different lifecycle stages. Additionally, the proportion of novel proteins (i.e. those proteins lacking homologs in other species) that were stage-specific was enriched compared to the total secretions (**Fig. 3 B**; HES 44.3% = 27/61, L4 ES 42.5% = 19/45, ERM 60.5% = 23/38). The vast majority of L4 ES proteins had a predicted signal peptide (92.5% = 198/214), greater than that seen for adult HES (79.7% = 290/364), whereas ERM was much lower (59.8% = 125/209), again suggesting that the latter contains constituents that are not actively secreted.

Two parallel approaches were taken to quantify the relative levels of proteins present in the different ES material and determine selective expression, protein abundance and gene expression profiling. Firstly, protein abundance was estimated by emPAI (exponentially modified Protein Abundance Index [31]), which provides an approximate protein ranking based on the number of observed peptides relative to the known full-length sequence. Because not all peptides are equally amenable to MS detection, and not all sequences are full length, this method is not infallible but provides a benchmark for further analysis and allows direct comparison of the relative abundance of an individual protein across different life-cycle stages.

**Figure 4:**
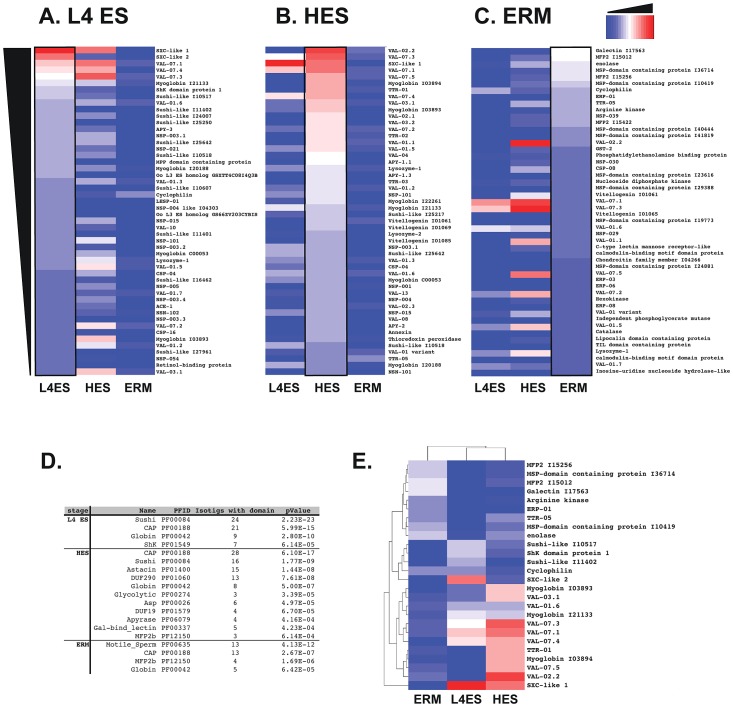
Comparison of protein abundance (emPAI) in each ES product. **A.** The 50 most abundant proteins in L4 ES, ranked by emPAI score (left column) with corresponding emPA scores for HES (centre) and ERM (right column). Scores are coloured on a log_2_ scale with red maximum and blue minimum. **B.** The 50 most abundant proteins in HES ranked as above (centre column) with corresponding scores for L4 ES and ERM. **C.** The 50 most abundant proteins in ERM ranked as above (right column) with corresponding scores for L4 ES (left column) and HES (centre). **D.** Protein family (Pfam) enrichment within each ES, ranked by statistical significance (cut-off of p<0.001) compared to Pfam in total transcriptomic assembly. “Isotigs with domain” indicates number of times domain present in indicated ES. Full analyses are provided in **Table S5**. **E.** Two-way clustering of the 10 most abundant proteins in the 3 ES preparations.

Heat maps ranking the top 50 highest emPAI-scoring L4 ES, HES and ERM are shown in **Figure 4 A–C** respectively, while complete alphabetical lists are presented in **Tables S1, S2, S3**. On the criterion of emPAI score, the most abundant L4 ES constituents were two members of the Shk/SXC gene family (PF01549) containing a conserved six-cysteine motif (SXC-like 1 and 2) and three isoforms of VAL-7 corresponding to 2D gel spots (**Fig. 1**). Most of these were also prominently expressed by the adult stage, although SXC-like proteins were highly upregulated in L4 ES. Members of the novel NSP-3 family were also expanded in the L4 stage. Most notably, a number of Sushi-domain containing proteins were relatively highly represented in L4 ES but absent from adult ES.

To form a broader picture of the functional pattern of each ES compartment, we also analysed the constituent proteins for domains corresponding to defined protein families in Pfam (**Fig. 4 D** **and Tables S4, S5**). Statistical comparison with Pfam domains detected in the total CDS of the *H. polygyrus* transcriptome (Harcus et al, manuscript in preparation) confirmed the selective release of protein families secreted by all parasite stages (e.g. PF00188 CAP indicating widespread release of VAL proteins). Furthermore, it also revealed regulation in the secretion of key gene families through parasite development (e.g. expansion of PF00084 Sushi and PF01549 ShK/SXC in L4 ES, and PF00635 motile_sperm in ERM). Two-way clustering of abundant proteins from the three ES preparations again shows close association in proteins released by L4 and adult worms, but not ERM, confirming the global similarity between larval and adult released material (**Fig. 4E**).

It is interesting to note that visual inspection of the 2D SDS-PAGE comparisons (**Fig. 1**) indicates a closer similarity between L4 and adult HES than emerges from the full LC-MS proteomic analysis, while ERM appears distinctly different. This reflects the relative abundance of the proteins shared by the L4 and adult stages. Moreover, a significant positive correlation was observed between the emPAI values of shared L4 ES and HES proteins (p<0.0001; Spearman r^2^ = 0.3478) but not between those of shared ERM and HES proteins (n.s.; Spearman r^2^ = 0.1660; **Fig. S1 A, B**); hence although certain proteins are present in both HES and ERM, their abundance may be very different. This is well illustrated by the heat map of emPAI values of the 60 proteins shared between all three ES preparations showing clear differences in expression level between the stages (**Fig. S1 C**). The identities and relative abundances of proteins shared between HES and L4 ES (which both include protective antigens), as well as HES and ERM (in which the latter is non-protective), are shown in **Fig. S1 D and E**.

### Transcriptomic analysis of ES protein-encoding genes

**Figure 5:**
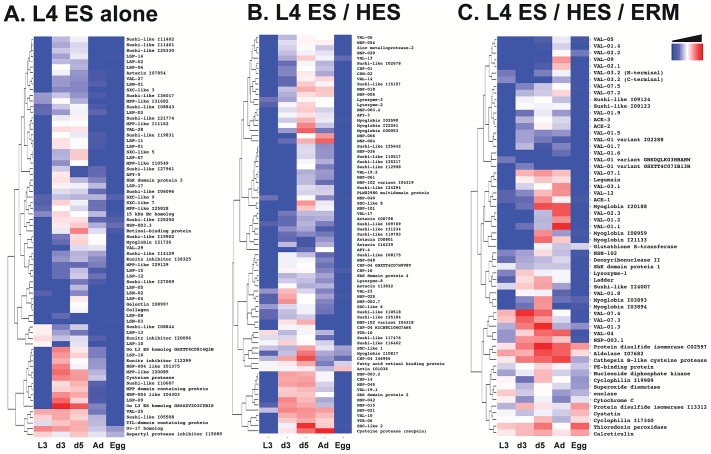
Gene expression (RPKM) heat-maps of stage-specific and shared ES proteins. **A.** L4 ES stage-specific proteins, clustered by gene expression profiles in a transcriptomic dataset based on 5 life cycle stages (infective L3, d3 post-infection L3, d5 post-infection L4, Adult, and Egg). RPKM = Reads Per Kilobase Mapped. Scores are coloured on a log_2_ scale with red maximum and blue minimum. **B.** As above for proteins shared between L4 ES and HES. **C.** As above for proteins shared between L4 ES, HES and ERM. The corresponding heat-maps for adult HES stage-specific proteins, and for ERM-specific proteins, are presented in **Suppl. Fig. 2 A and B** respectively.

An independent method of determining the stage-specific expression of ES proteins was based on an extensive transcriptomic database, using a normalised read count (RPKM; reads per kb per million mapped reads [32]) to create a heat map corresponding to relative gene expression across the five different lifecycle stages (**Fig. 5 A–C**). This revealed most L4 and adult ES proteins are encoded by genes that are relatively quiescent in the free-living L3 stage, and transcription is either initiated or upregulated following host entry. Generally, gene expression analysis supports the proteomic data; expression of L4-specific ES proteins is mostly at its highest in day 3 or 5 larvae, before downregulation in the adult (**Fig. 5 A**), expression of shared L4 ES and HES proteins is maintained in day 3, 5 and adult worms (**Fig. 5 B**), and expression of HES-specific proteins is highest in adult worms (**Fig. S2 A**). The expression of the “core 60” ES proteins common to L4 ES, HES and ERM splits into two main clusters, those that are expressed by all 5 stages and those mainly expressed by d3, d5 and adult worms (**Fig. 5 C**). A small cluster of ubiquitously expressed genes was also seen in ERM-specific proteins, but surprisingly the majority of ERM-specific proteins were not actually expressed by the egg, and instead must be derived from the adult worm where expression is greatest (**Fig. S2 B**). This includes sperm-derived proteins present in ERM, such as the MSP-domain proteins (13×) and MFP2 (4×), as well as the egg sac vitellogenins (5 variants).

Comparison of protein abundance (emPAI) with gene expression (RPKM) for all L4 ES proteins showed that protein levels positively correlate with gene expression of day 5, but surprisingly not day 3, larvae (**Fig. S3 A–E**). This reflects the major changes in gene expression either side of the L3 to L4 molt, with new transcription of many new genes being initiated (Harcus Y. *et al*, manuscript in preparation). Notably, the significant correlation between protein emPAI and mRNA RPKM within the L4 stage lends further validation to these parameters as quantitative measures of expression in the nematode worm.

### Predominant and enriched protein families in L4 ES

**Figure 6:**
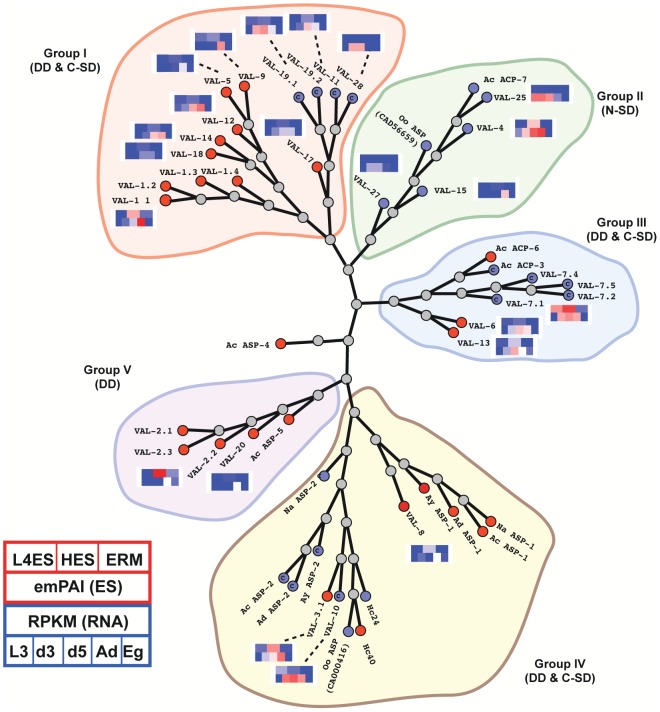
Phylogeny and expression of VAL family members in *H.polygyrus*. Phylogenetic tree of *H. polygyrus* secreted VAL proteins and those from selected other intestinal nematodes (Na = *Necator americanus*; Ac = *Ancylostoma caninum*; Ad = *Ancylostoma duodenale*; Ay = *Ancylostoma ceylanicum*; Oo = *Ostertagia ostertagi*; Hc = *Haemonchus contortus*). Blue nodes represent single SCP domain VAL/ASP proteins whereas red nodes indicate double SCP domain proteins; blue nodes are indicated as more similar to N-terminal (N) or C-terminal domains (C) as appropriate. Protein (emPAI, top in same order as Fig. 4) and gene (RPKM, bottom, in same order as Fig. 5) expression profiles are indicated for *Hp* VAL proteins. Expression profiles are for most abundant variant where more than one is present. *H. polygyrus* VAL-21 to 24 and VAL-29 have been omitted as their sequences were either incomplete are did not unambiguously align with a single branch of the tree. On the basis of this phylogenetic analysis, 5 groups of VAL proteins are indicated. Note that this does not conform with a 3-group classification recently proposed [69] as we found the Groups 1 and 3 defined by these authors to be polyphyletic.

The most highly represented proteins in both L4 and adult ES are VAL (Venom allergen/*Ancylostoma* secreted protein-Like) family members; these are part of a larger CAP protein superfamily (Pfam PF00188), which includes mammalian sperm-coating protein (SCP) that lends its name to the canonical domain structure. The CAP superfamily shows extensive diversity in most species, with over 30 genes in mice and humans [33]; in this respect, *H. polygyrus* is not exceptional in expressing over 20 different VAL family members (numbered in descending order of abundance in adult HES) although their representation differs between adult and L4 ES. Generally, L4 ES contains lower levels of most VAL proteins compared to HES, with just trace amounts of VAL-2 present, and there is no abundant L4 ES VAL protein not previously described in adult HES. Only the low abundance VAL proteins VAL-23, 25, 27, 28 and 29, as well as the moderately abundant VAL-10, are enriched in L4 ES compared to HES, a pattern replicated at the transcript level for the low abundance VAL proteins (**Fig. 6** **and Table S1**).

Using specific mAb, we confirmed by ELISA that L4 ES contains little VAL-2, but that other abundant VAL proteins (VAL-1, 3 and 4) are present at levels similar to adult HES (**Fig. S4 A–D**). In contrast, little VAL-1 was detected in ERM (**Fig. S4 A**) despite LC-MS analysis showing the presence of several variants (**Table S3**). This is suggestive of partial degradation, consistent with the 2-D gel of ERM lacking the characteristic stretch of VAL-1 isoforms readily identifiable in both HES and L4 ES (**Fig. 1 A–C** and [25]).

As has been noted in studies with other helminth species [34], [35], the VAL gene family shows extensive evolutionary diversity within *H. polygyrus*, in both sequence and domain structure (**Fig. 6**). Additionally, proteins expressed in a stage-regulated manner do not form a clear structural subset within this family as within Group 1 alone (**Fig. 6**) VAL-1 is preferentially expressed by the adult, VAL-28 is restricted to the d3/d5 larval transcriptome, whereas others (such as VAL-12) show a broader pattern of expression. Such contrasting profiles may reflect fine adaptation of different family members to specialised functional roles. Notably, comparison with the closely related *Ancylostoma* and *Necator* hookworm species reveals an intricate pattern of gene expansion and contraction: Group 1 *H. polygyrus* VAL proteins are clearly separated from the human and dog hookworm ASP-1-like VAL-8, ASP-2-like VAL-3 and ASP-5-like VAL-2. Hence, adaptation to the murine host may have resulted in an accelerated radiation of VAL family members.

While the VAL family is less prominent in L4 ES than HES, other protein families are distinguished by maximal representation in the L4 stage. These include two conserved families, the Sushi-like proteins and the ShK/SXC-like proteins, as well as a newly defined set of Novel Secreted Protein-3 (NSP-3) variants.

Sushi domains (PF00084) are 60-amino acid consensus sequences with 4 conserved cysteine residues, and are prevalent in mammalian proteins that regulate complement activation, hence the alternative domain name of complement control protein (cd0003; [36]). Of note, Sushi domains in complement regulatory proteins generally occur in repeated units (e.g. complement receptor 1A, 30 repeats; factor H, 20 repeats), which is also evident in an *Ascaris* complement factor H homolog (15 repeats; ADY39830.1) and similar proteins in *Loa loa* (12 repeats; XP_003143211.1) and *C. elegans* (11–16 repeats; Ce F36H2.3 isoforms A-G). Some 34 L4 ES proteins contained sequences related to the Sushi domains (pfam 00084), which we have collectively termed Sushi-like proteins as detailed in **Fig. S5**. Of these, 18 are also present in HES albeit at lower levels, together with an additional 5 not present in L4 ES. ERM contained only two Sushi-like proteins at low levels. The majority of *H. polygyrus* sushi-like proteins however, have only 1 or 2 sushi domains (**Fig. S5 A**) more similar to those of cytokine receptors such as the alpha subunits of the IL-2 (2 domains) and IL-15 (1 domain) receptors [37].

ShK/SXC proteins have a 36 amino acid domain with 6 conserved cysteine residues, and are widely expressed by parasitic and non-parasitic nematodes [38], [39]. L4 ES contains 15 proteins with ShK domains, with an additional one detected at low levels in HES. Several of these domains (7/16) were fused to other sequence motifs; 3 astacin metalloproteases and 4 homologs of hypothetical (non-annotated) *C. elegans* proteins. In the latter cases, proteins contained either 2 or 4 tandemly repeated ShK domains (**Fig. S6 A**), in a pattern very similar to that observed in other parasitic nematodes, such as *T. canis* mucins [39]. More unusually, many ShK proteins (9/16) in *H. polygyrus* ES are found as short single-domain proteins (full-length 63–67 aa, mature protein 41–49 aa) consisting of no more than a signal sequence and the 6-cysteine domain (**Fig. S6 B**). As this is reminiscent of *Ostertagia ostertagi* SXC-1 (accession number CAC17797), we have termed these proteins SXC-like. The parasite appears primed to produce this group of proteins even before infection of the mammalian host as gene expression is detected for 5/9 in the L3 stage, and peaks in day 3–5 larvae.

L4 ES contains relatively high levels of several enzymes including apyrases (particularly APY-3, but also low levels of a new L4-specific APY-5), lysozymes and the three acetylcholinesterase proteins (ACE-1, 2 and 3) previously identified in adult secretions [25]. ACE levels in L4 ES were comparable to those in HES, as determined by emPAI, an expression pattern confirmed by RPKM transcript analysis, which revealed low or absent expression in L3 and eggs, and peak expression by day 5 L4 parasites, maintained in adults (**Tables S1, S2, S3**). In contrast, significant products which are preferentially secreted by the luminal adults, and which have previously been reported to be secreted by parasitic nematodes, include proteases such as astacins and zinc metalloproteases [40]–[42], cathepsins [43], [44] and trypsin-like and other serine proteases [45]–[47], serpin [48] and Kunitz protease inhibitors [49], and predicted chitinases and chitin-binding proteins [50].

In common with most nematode transcriptomic analyses, a large proportion of expressed protein genes either lack identifiable homology to proteins of other species, or only match hypothetical *C. elegans* proteins. These included over 80 distinct sequences incorporating a canonical signal peptide and found in either or both L4 ES and HES (**Table S1**). Those unique to L4 ES were designated Larval Secreted Proteins (LSPs) while those present in HES adhered to the earlier designation of Novel Secreted Proteins (NSPs). Some 17 distinct LSPs were identified, while a further 17 L4 ES proteins were among a total of 50 NSPs present in the adult HES. The L4-specific proteins LSP-1 and LSP-2 were similar to the adult-specific ES constituents NSP-28 and NSP-53, indicating micro-adaptation of sequences to the different niches. Similarly, seven closely related variants of NSP-3 were also identified, one of which was found only in the L4 ES and two other only in HES. A further grouping was formed by three L4 ES NSP-4-like proteins, with homology to adult secreted NSP-4, 6, 10, 12, 18 and 44.

Adult HES contains an as yet unidentified TGF-β -like molecule that is able to directly induce Foxp3^+^ regulatory T cells in the presence of a mitogenic stimulus [51]. Using a TGF-β reporter cell line, L4 ES was also shown to possess TGF-β activity (59.4±14% of the level present in adult HES over multiple batches), whereas this was undetectable in ERM (**data not shown**). Scrutiny of the mass spectrometric data did not reveal any peptides matching either mammalian TGFβ, or members of the TGF-β superfamily previously identified within the genome and transcriptome of *H. polygyrus* [52]. Hence, it is possible that the ability of HES to ligate the mammalian TGF-β receptor is mediated by one of the novel secreted proteins described above.

### Protective and non-protective antigens following ES immunisation

**Figure 7:**
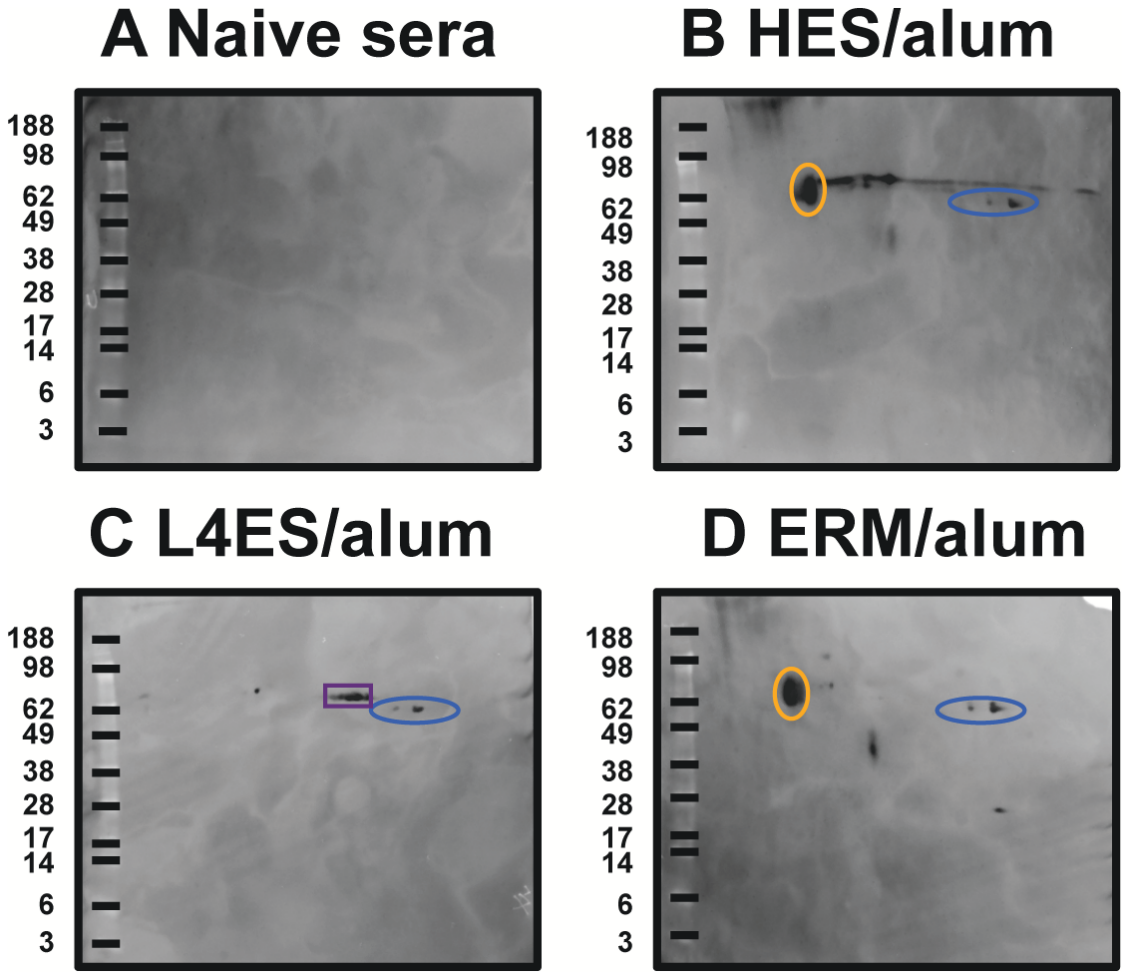
ERM immunization elicits class-switched anti-glycan B antibodies. **A–D** Western blot analysis of IgG1 antibodies from (A) naïve, or pre-challenge (B) HES, (C) L4 ES and (D) ERM immunised mice. Spots corresponding to VAL-1/glycan A (blue), glycan B (orange) and ACE-1 (purple) are shown. Molecular weight markers (kDa) are indicated.

We noted earlier that ERM induced similarly high titers of anti-HES IgG1 antibodies to those generated by protective L4 ES or HES immunisation (**Fig. 2 C**) while failing to induce immunity, suggesting that non-protective but cross-reactive epitopes were well represented in these antigen preparations. We have previously identified two non-protective immunodominant carbohydrate epitopes in *H. polygyrus* with monoclonal antibodies, namely Glycan A, an O-linked sugar attached to several VAL proteins (including VAL-1), and glycan B, a proteoglycan-like structure abundant in the adult worm soma [27]. Using the same monoclonal antibodies, we found that whilst L4 ES contained markedly less of both Glycans A and B than adult HES, confirming previous work [27], ERM contained substantially more glycan B than the other preparations (**Fig. S4 E–F**). Consistent with this, Western blot analysis revealed serum IgG1 from HES and ERM, but not L4 ES, immunised animals showed strong reactivity to a 65 kDa spot representative of glycan B (**Fig. 7 A–D**). All three preparations induced IgG1 reactivity against VAL-1 spots, suggesting class-switched antibodies binding to glycan A. Additionally L4 ES immunisation resulted in Western reactivity to spots corresponding to ACE-1 (**Fig. 7 C**).

**Figure 8:**
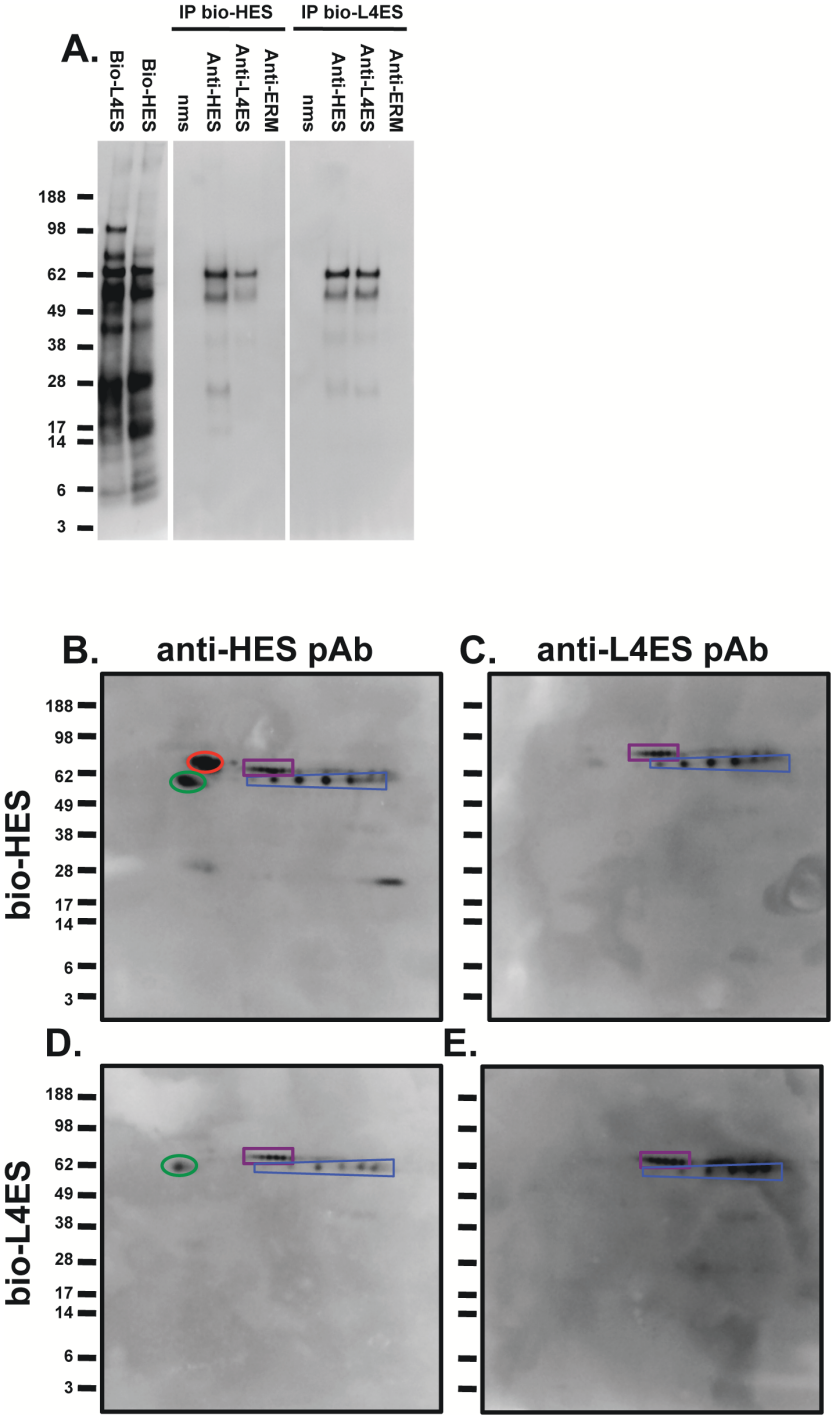
HES and L4 ES immunization both induce recognition of VAL-1 and ACE-1. **A.** 1-D immunoprecipitation (IP) of biotin-labelled HES (Bio-HES) and L4 ES (Bio-L4ES) with naïve mouse serum (nms) or sera from mice vaccinated with HES, L4 ES or ERM taken immediately before challenge with live larvae. Total biotin-labelled L4 ES and HES are included for comparison. **B–E.** 2-D immunoprecipitation of biotin-labelled HES (**B–C**) and L4 ES (**D–E**) with anti-HES (**B, D**) or anti-L4 ES (**C, E**). Spots corresponding to VAL-1 (blue), VAL-2 (red), VAL-3 (green) and ACE-1 (purple) are shown. Molecular weight markers (kDa) are indicated.

To identify potential protective protein antigens, we analysed immunised sera through an immunoprecipitation protocol which captures responses to conformation-dependent protein epitopes [27]. Immunoprecipitation of biotin-labelled ES with sera from immunised mice revealed a more extensive profile of targeted antigens, with sera from L4 ES and HES immunised mice binding to a range of proteins in both L4 ES and HES (**Fig. 8 A**). In contrast, ERM immunisation did not elicit such antibodies, providing further evidence that the high anti-HES IgG1 titers generated bind almost exclusively to non-protective cross-reactive glycans. On 2-D analysis, it was found that while HES immunisation generated strong antibody recognition of VAL-1, 2 and 3, as well as ACE-1, L4 ES only induced antibodies against VAL-1 and ACE-1, and these appeared responsible for the vast majority of the observed immunological cross-reactivity between the two preparations (**Fig. 8 B–E**). These data argue that the minimal requirement for protective immunity is recognition of a single VAL protein (VAL-1) and ACE-1.

## Discussion

The larval and adult stages of parasitic nematodes often colonise different physiological niches. Distinct immune pressures exerted in each environment will likely be reflected in the preferential expression of key genes and protein families capable of modulating host immunity. In this regard, the tissue-phase ES material of the intestinal nematode *H. polygyrus* was characterised by increased expression of proteins associated with the regulation of complement (sushi-like) and T cell activation (SXC-like), as well novel proteins of unknown function (e.g. NSP-3 family). However, other proteins are common to both L4 ES and HES, and it is two of these (VAL-1 and ACE-1) that are major antigenic targets in mice rendered fully immune to challenge infection by vaccination with either of these preparations.

We originally hypothesised that protective immunity induced by ES immunisation would function by neutralising parasite immunomodulatory molecules. Although no common mechanistic function has yet been attributed to VAL proteins or the CAP superfamily, it is notable that they are most prominent at interfaces between organisms (virally-infected plants, insect venoms, helminth parasite secretions and the mammalian male gamete) and in some instances are associated with immunological down-modulation [53], [54]. Alternatively, immunisation may inhibit proteins (such as ACE-1) with key but uncharacterised roles in parasite metabolism, migration, and coordination. It is also possible that vaccine-elicited antibodies protect through their Fc domains by recruiting innate immune cells to kill the invading larvae, hypotheses we are currently testing. Potential immunomodulatory roles can also be ascribed to the Sushi and SXC-like proteins. Homologs of sushi proteins from vaccinia viruses are able to inhibit both the classical and alternative pathways of complement [55], whereas a SXC protein homolog from the sea anemone *Stichodactyla helianthus* was shown to block mammalian potassium channels, and hence was named ShK [56]. ShK proteins and related sequences can inhibit calcium-dependent lymphocyte activation [57], suggesting a direct immunomodulatory role for ShK homologues from nematode parasites.

Helminth pathogens regulate their physiological and immunological environment through a spectrum of released products that are being characterised by new high throughput technologies. Our study on the mouse model *H. polygyrus* directly complements many datasets with major human pathogens such as *Brugia malayi* [58]–[62] and the hookworm species [63], [64], as well as economically important trichostrongylid nematodes [65]–[67]. Each of these parasites has evolved specialised strategies adapted to particular host species, and niches within those species, that will be mirrored in the repertoire of secreted products, and our study provides many examples of protein families which are differentially expressed, expanded or regulated during the course of a parasite life cycle. Importantly, in view of the lack of anti-helminth vaccines for human use, we have also tested ES products from larval and adult stages for their ability to induce sterilising immunity and in combination with the proteomic database, have now identified potential vaccine antigens for future appraisal. Taken together, these analyses have defined an extensive and novel repertoire of protein candidates from *H. polygyrus* that can be taken forward for functional and immunological testing in a tractable and naturally adapted host-parasite model system.

## Materials and Methods

### Ethics statement

All animal protocols adhered to the guidelines of the UK Home Office, complied with the Animals (Scientific Procedures) Act 1986, were approved by the University of Edinburgh Ethical Review Committee, and were performed under the authority of the UK Home Office Project Licence number 60/4105.

### Parasite maintenance and preparation of ES material

CBAxC57BL/6F1 mice were infected by gavage with 500 L3 stage larvae of *H. polygyrus bakeri* (originally provided by Professor J M Behnke, University of Nottingham, UK). Day 5 4^th^ stage larvae and day 14 adults were washed, cultured in serum free media and the ES concentrated as before [25], [27]. For egg released material (ERM), *in vitro* released eggs from adult worms were washed and cultured for 3 days in serum free media before concentration and diafiltration into PBS using 5000 MWCO spin columns (Vivaspin). L4 produced 10±2 ng ES protein/larva/day, compared to adult worm production of HES at 18±2 ng/adult/day. Eggs cultivated for 3 days *in vitro* released 56±15 pg protein/egg/day. Somatic extracts of L4 and egg were prepared with a Qiagen tissuelyser.

### 2-D gel electrophoresis, LC-MS/MS and bioinfomatics

L4 ES, adult HES, ERM, L4 extract and egg extract (10 µg) were separated by 2-D gel electrophoresis, protein spots of interest excised, trypsinised and analysed by MALDI, or trypsinised and assessed by LC-MS/MS as previously described [25], [27]. MS spectra were submitted to a locally running copy of Mascot (v2.3, Matrix Science) and searched against an improved in-house BLASTx annotated database of >57K isotigs and >92K singletons obtained by 454 sequencing of *H. polygyrus* infective L3, day 3 larvae, day 5 larvae, adults and eggs, with additional full length *H. polygyrus* sequences from NCBI and our own Sanger sequencing (Harcus Y. *et al*, manuscript in preparation). Search parameters required trypsin specificity, cysteine modification, possible oxidation of methionine, and allowed one missed cleavage. Gel spot identifications had a peptide tolerance of 100 ppm and MS/MS tolerance of 0.5 Da, with peptide expect scores <0.05. MudPit scoring was used for LC-MS with a <0.05 significance threshold. Single peptides were more stringently filtered for expect values <0.01. Each protein had at least one peptide not present in higher ranked hits and was manually inspected for open reading frames. Peptide false discovery rates were estimated using a decoy database (0.88% L4 ES; 2.18% HES; 1.5% ERM). Identified ORF were assessed for protein domains (Pfam v26.0) and N-terminal signal peptides (SignalP v4.0). The statistical significance of Pfam domain enrichment was determined for selected subsets using the hypergeometric functions of the “Category” Bioconductor package. Protein abundance was estimated by emPAI (exponentially modified protein abundance index [31] which calculates the ratio of observed∶observable peptides. Spectral counts were also included for comparison. Sequence alignments and phylogenetic trees were carried out with MacVector (v11.1.1) and COBALT (NCBI). Heat maps and sequence clustering were generated with Artemis (Wellcome Trust Sanger Institute). When analysing stage-specific expression, proteins were treated as isogroups (i.e. closely related micro-variants of the same protein, likely representing alleles), meaning proteins were classed as shared even if the specific isotig was not detected (“shared isogroups”). Such analysis ensured that the determination of stage-specific protein expression was not skewed by the presence or absence of closely related alleles, detection of which likely depends on the relative abundance of the protein in the sample rather than any biological significant difference.

### Vaccinations

C57BL/6 female mice were immunized with 5 µg L4 ES, HES, ERM or PBS control i.p. in alum adjuvant, boosted on days 28 and 35 with 1 µg in alum i.p., before oral challenge with 200 *H. polygyrus* L3 at day 42. Fecal egg counts were determined at days 14, 21 and 28 post-challenge, and adult worms counted at day 28.

### ELISAs, Western blots and IP

Serum was obtained by tail bleeds from vaccinated mice immediately prior to challenge and assessed for anti-L4 ES, HES or ERM IgG1 reactivity as described before [27]. VAL-1, 2 3, and 4, glycan A and B levels in the different ES preparations was determined using specific mAb obtained from primary infection (anti-VAL-1, 4-M15; anti-VAL-2, 4-S4; anti-VAL-4, 2–11; anti-glycan A, 13.1), secondary infection (anti-VAL-3, 5-S1) and HES immunisation (anti-glycan B, 9.1.3) ([27] and Filbey K.J *et al*, manuscript in preparation]. Western blotting of unlabelled HES, biotin-labelling and IP of ES material, followed by blotting and strep-HPO visualisation was carried out as before [27].

### TGF-β bioassay

MFB-F11 cells [68] were cultured as before [51] ±20 µg/ml ES material or with varying amounts of rhTGF-β1 standard (R & D systems). After 24 hours, supernatants were collected and alkaline phosphatase activity determined with SEAP reporter assay kit (InvivoGen) as per manufacturer's instructions.

### Statistical analysis

Statistical significance was determined by ANOVA or Mann-Whitney test where indicated. Correlation was measured with Spearman's rank as emPAI values are non-parametric. All statistical analysis were performed using Prism (v6.0)

## Supporting Information

Figure S1 **L4 ES protein composition is more similar to HES than is ERM.** **A.** Comparison of emPAI values for proteins shared between L4 ES and HES. Spearman r values indicate correlation coefficients, line indicates linear regression (*** = p<0.001). **B.** As above for proteins shared between ERM and HES (n.s. = non-significant). **C.** Heat map of emPAI values for core 60 proteins shared between L4 ES, HES and ERM indicating differential expression. **D.** As above, for L4ES-HES shared proteins. **E.** As above, for HES-ERM shared proteins.
(EPS)

Figure S2 **Gene expression (RPKM) heat-maps of HES-specific and ERM proteins.** **A.** HES -specific proteins, clustered by gene expression profiles in a transcriptomic dataset based on 5 life cycle stages (infective L3, d3 post-infection L3, d5 post-infection L4, Adult, and Egg). RPKM = Reads Per Kilobase Mapped. Scores are coloured on a log_2_ scale with red maximum and blue minimum. **B.** As above for proteins found only in ERM.
(EPS)

Figure S3 **Stage-specific gene expression of L4 ES proteins.** Comparison of emPAI values of L4 ES proteins with RPKM gene expression levels from: **A.** Infective stage L3 larvae **B.** Day 3 post-infection L3 larvae **C.** Day 5 post-infection L4 larvae **D.** Adult worms **E.** Eggs. Spearman r values indicate correlation co-efficients (*** = p<0.001; n.s. = non-significant).
(EPS)

Figure S4 **A. Levels of VAL-1 in L4 ES (blue), HES (red) and ERM (yellow) determined by reactivity with the mAb 4-M15** [**27**] **.** **B.** As above for VAL-2 (mAb 4-S4). **C.** As above for VAL-3 (mAb 5-S1). **D.** As above for VAL-4 (mAb 2–11). **E.** As above for Glycan A (mAb 13.1). **F.** As above for Glycan B (mAb 9.1.3).
(EPS)

Figure S5 **Sushi-domain proteins.** **A.** Phylogenetic tree of Sushi-domain containing proteins in L4 ES and HES. Blue domains indicate significant Pfam matches (E-value<0.01) to Sushi domain (pf00084); green domains indicate lower level similarities (E-value 0.01–0.05) retaining recognisable homology to pf00084. Signal peptides are depicted in yellow, presumed N-terminal truncations by broken lines, and stretches of >50 amino acids without detectable homology are indicated by black bars. **B.** Heat maps showing protein (emPAI) and transcript (RPKM) expression of indicated sushi-like proteins.
(EPS)

Figure S6 **ShK/SXC-like proteins.** **A.** Cartoon indicating domain structure of ShK/SXC-like proteins (top) compared to other ShK domain proteins (bottom). ShK domain indicated by orange, N-terminal signal sequence by yellow, astacin domains by purple and presumed N-terminal truncation by broken lines. Heat maps showing protein (emPAI) and transcript (RPKM) expression of indicated proteins also shown. **B.** Sequence alignment of ShK/SXC-like proteins indicating mature protein following removal of N-terminal signal peptide. Positions of the canonical 6 conserved cysteine residues are indicated in yellow.
(EPS)

Table S1 **Full A-Z list of proteins identified in L4 ES.** “emPAI rank” represents ranked abundance, “spectral count” is total number of peptides mapped to the protein of interest, whereas “peptides” is the number of different peptide sequences detected. “SS?” shows +/− N-terminal signal sequence, with * indicating the sequence is N-terminally truncated but its closest BLAST homolog is SS+ve. emPAI values for L4 ES, HES and ERM are indicated, as are RPKM transcript levels for L3, day 3, day 5, adult and egg. Proteins present only as “shared isogroups” (see materials and methods) are shared grey. One protein previously identified in HES, CSP-4, is present in 3 fragments in the transcriptomic assembly, and is only counted once.
(XLSX)

Table S2 **Full A-Z list of proteins identified in HES.** As above for HES.
(XLSX)

Table S3 **Full A-Z list of proteins identified in ERM.** As above for ERM.
(XLSX)

Table S4 **Full list of Pfam domains in different ES preparations.** Pfam domains present in ES proteins listed by expression pattern. emPAI and RPKM values included to show relative expression.
(XLSX)

Table S5 **Enrichment of Pfam domains in different ES preparations.** Pfam domains present in ES proteins ranked by statistical significance compared to their frequency in the *H. polygyrus* transcriptome assembly.
(XLSX)
